# Supplementary material for: Strain-induced long-range charge-density wave order in the optimally doped Bi2Sr2−xLaxCuO6 superconductor
Source: Nat Commun. 2024 Jun 14;15:5082. doi: 10.1038/s41467-024-49225-w (PMC11178839; doi:10.1038/s41467-024-49225-w)
Supplement: Supplementary file 1 — Supplementary Information [file 41467_2024_49225_MOESM1_ESM.pdf]

Supplementary Information for

Strain-induced long-range charge-density wave order in the optimally doped $Bi_2Sr_{2-x}La_xCuO_6$ superconductor

Shinji Kawasaki[1], Nao Tsukuda[1], Chengtian Lin[2], and Guo-qing Zheng[1]

[1]*Department of Physics, Okayama University, Okayama 700-8530, Japan*
[2]*Max-Planck-Institut fur Festkorperforschung, Heisenbergstrasse 1, D-70569 Stuttgart, Germany*

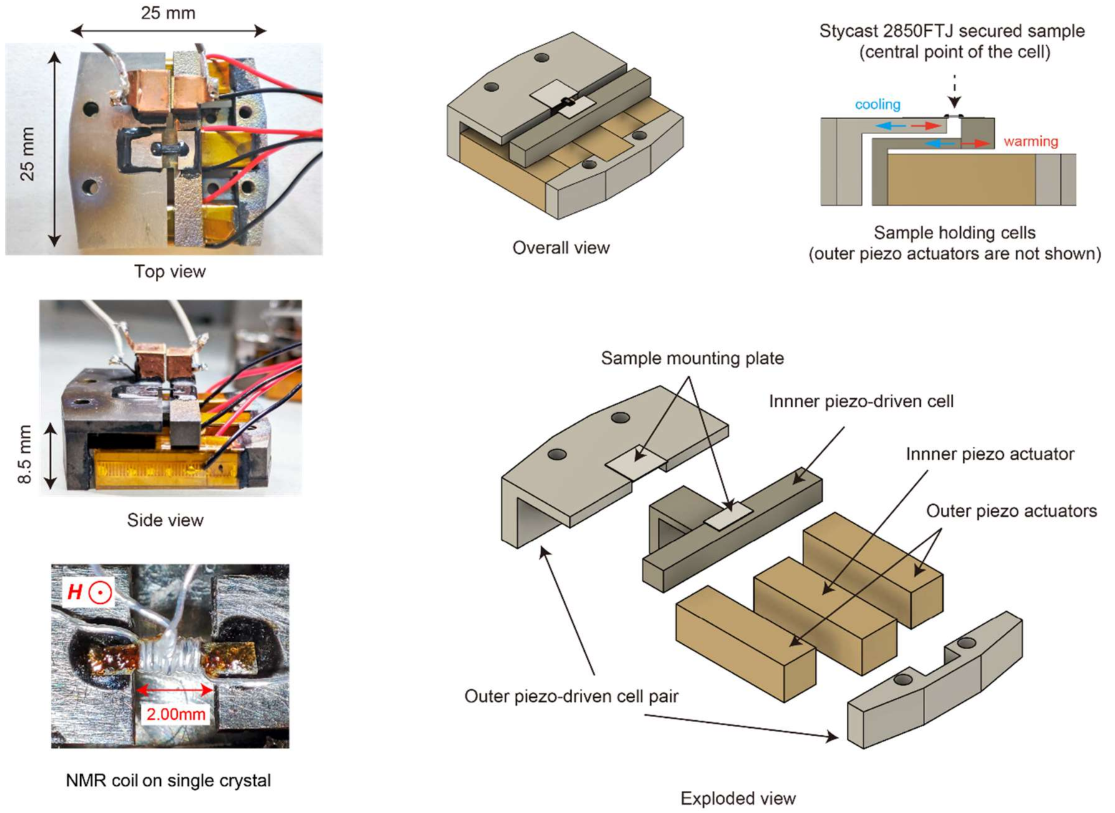

Supplementary Figure 1 | Homemade strain cell. Our strain cell apparatus.

Supplementary Note 1

Supplementary Figure 1 shows our homemade piezo-driven strain cell inspired by Hicks's cell [79]. The cell is assembled by bonding three cells and three commercially available piezoelectric actuators (PI, P-885.51). Three cells were cut out of a pure titanium (Ti) disk (The Nilaco Corporation, 99.5%) by electric discharge machining at the machine shop of Okayama University. Two actuators were embedded in the outer cell pair and one actuator in the inner cell. Epoxy (Stycast 2850FTJ) was used for bonding. The holes are 2 mm screw holes.

By centering the sample in the cell for NMR experiments, our cell design successfully minimizes potential residual stress that may occur in the sample at low temperatures. This is achieved by ensuring that the thermal contraction of the outer and inner cells remains in phase during temperature changes. The Ti-sample mounting plate can be replaced according to experimental needs such as sample size. The homemade parallel-plate capacitor fixed on the cell has a gap of less than 0.1 mm (about 1.5 pF at room temperature).

The sample is fixed as follows; a thin layer of thoroughly degassed epoxy is applied to a sample mounting plate. The sample is then placed and an epoxy is dropped onto the sample. The droplet is then shaped symmetrically. A PTFE-coated Ag wire NMR coil is directly wound on the paraffin paper, which is placed on top of the sample-holding epoxy with GE7031 varnish. A magnetic field $H^{\parallel c}$ = 13 T is applied perpendicular to the strain direction of the sample.

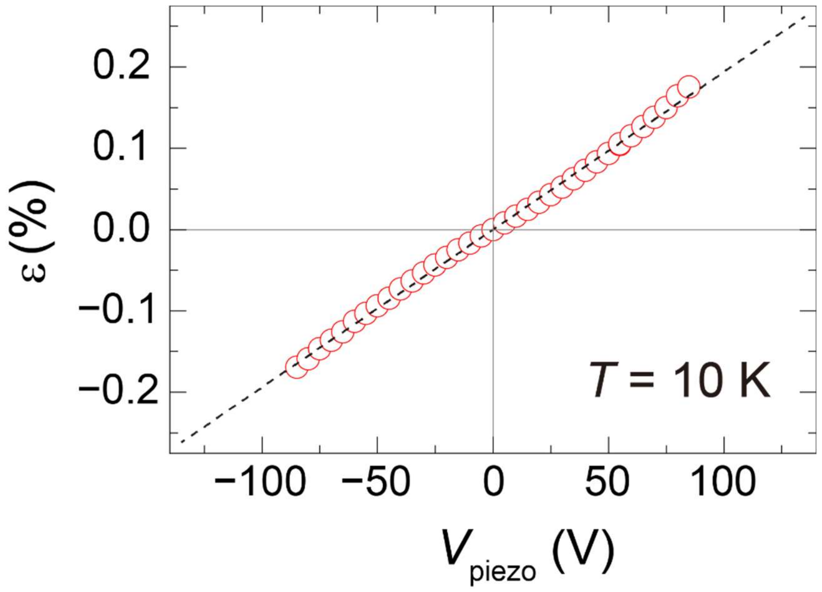

Supplementary Figure 2 | Evidence of elastic deformation in a single crystal plate. The applied voltage and strain in the NMR experiment at T = 10 K. The dashed straight line shows that the strain and applied voltage are proportional as $\varepsilon = 1.94 \times 10^{-3}\, V_{\mathrm{piezo}}$.

Supplementary Note 2

To ensure that the single crystal plate is undergoing elastic deformation, the applied voltage on the actuators and sample strain measured via a nearby parallel-plate capacitor are monitored at each temperature during the application of strain. Supplementary Fig. 2 shows an example.

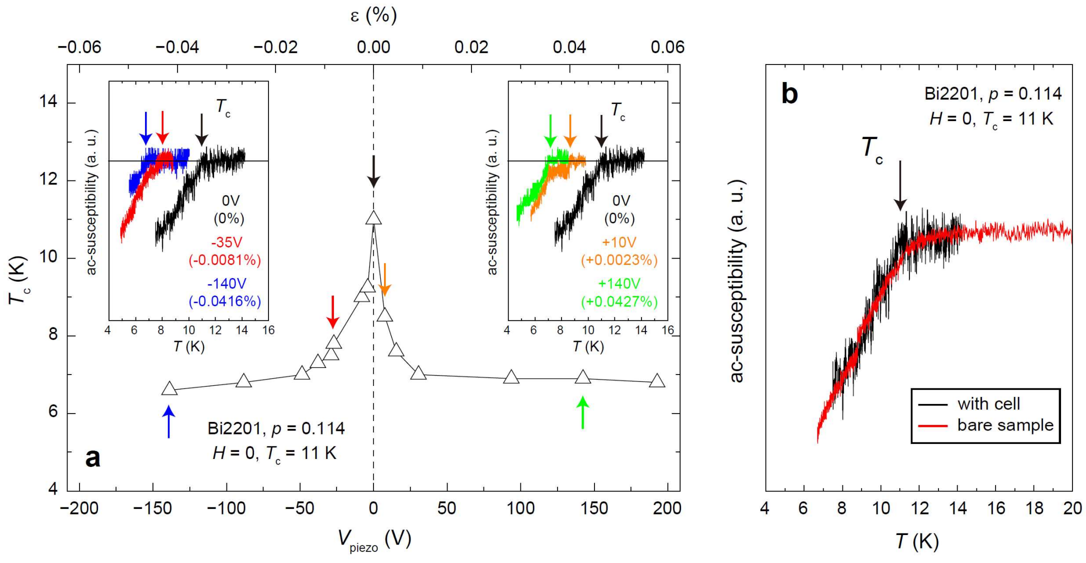

Supplementary Figure 3 | Negligible residual strain in the sample at low temperature. a T_c vs. the voltage applied on actuators (V_{piezo}) for underdoped Bi2201 superconductor (p = 0.114, T_c = 11 K) at H = 0. Insets: temperature dependence of the ac-susceptibility under compressive (left) and tensile (right) strain. **b** Temperature dependence of the ac-susceptibility with cell [fixed on the cell (V_{piezo} = 0)], and bare sample [without the cell (zero strain)].

Supplementary Note 3

Supplementary Figure 3a shows external strain dependence of T_c of the underdoped Bi2201 superconductor (p = 0.114, T_c = 11 K) at H = 0 (unpublished data). The T_c exhibits a significantly greater sensitivity to external strain compared to the optimally doped Bi2201 superconductor. Supplementary Figure 3b shows no significant difference in T_c between the sample with cell at V_{piezo} = 0 and without cell (bare sample). This result suggests that the residual strain arising from thermal contraction is negligible. Therefore, V_{piezo} = 0 can be considered to correspond to zero strain at low temperatures. The difference in signal to noise ratio is attributed to the difference in the size of the single-crystal samples used in the measurements.

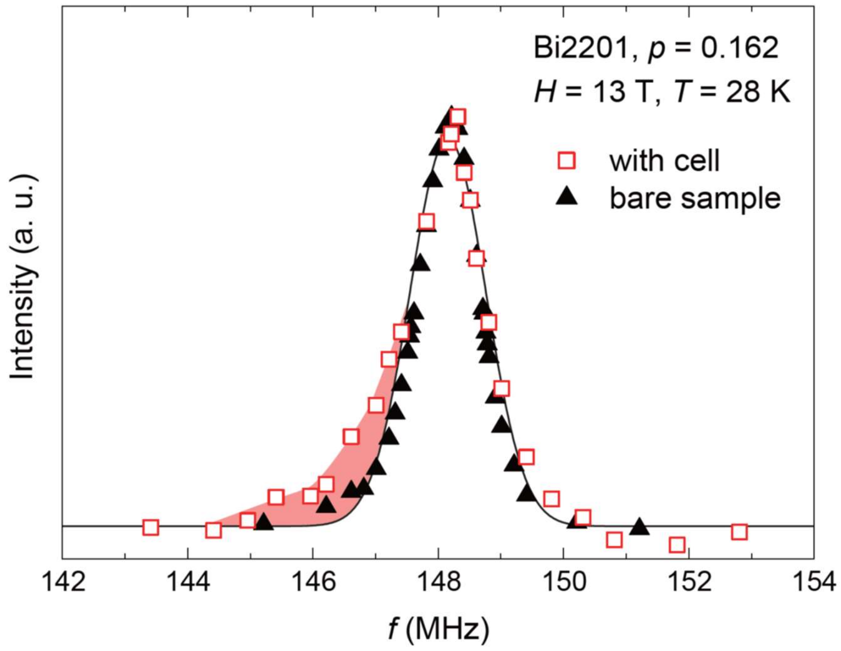

Supplementary Figure 4 | ^{63}Cu-NMR spectra with and without strain cell. The ^{63}Cu-NMR center line of a Bi2201 (p = 0.162) single crystal plate from the same batch measured at H = 13 T and T = 28 K. Open squares and solid triangles denote data from the crystal fixed on the strain cell (with cell) and the bare crystal (without cell), respectively. An additional ^{63}Cu-NMR signal, indicated by the shaded region, originates from the strain cell. The solid curve represents a Gaussian fit for the with cell spectrum, but the fitting range is restricted to frequencies above f = 147 MHz.

Supplementary Note 4

Supplementary Figure 4 shows the ^{63}Cu-NMR center spectrum measured with or without the strain cell. The signal in the shaded area of the spectrum with cell is absent in the spectrum of the bare sample (without cell). This suggests it originates from the copper in the parallel plate capacitor (made of copper and brass) placed near the sample, not from the sample itself. In a copper metal, the Knight shift (K) is known to be 0.23% (corresponding to 146.5 MHz at 13 T). Hence, to isolate the sample's contribution and accurately determine the Knight shift and full-width at half maximum (FWHM) using a Gaussian fit (represented by the solid curve), we utilized only data points above f = 147 MHz, where this non-essential signal is negligible. For example, Supplementary Fig. 4 indicates the obtained peak frequency (f_c) at T = 28 K is f_c = 148.169 MHz. From the relation $K = \frac{f_c - \gamma H_0}{\gamma H_0} \times 100 = \frac{148.169 - 11.285 \times 12.951}{11.285 \times 12.951} \times 100$, K =1.37 % is obtained. Since K_{orb} = 1.21 % [12], and thus, $K_s = K - K_{orb}$ = 1.37 −1.21 = 0.16 % is determined.

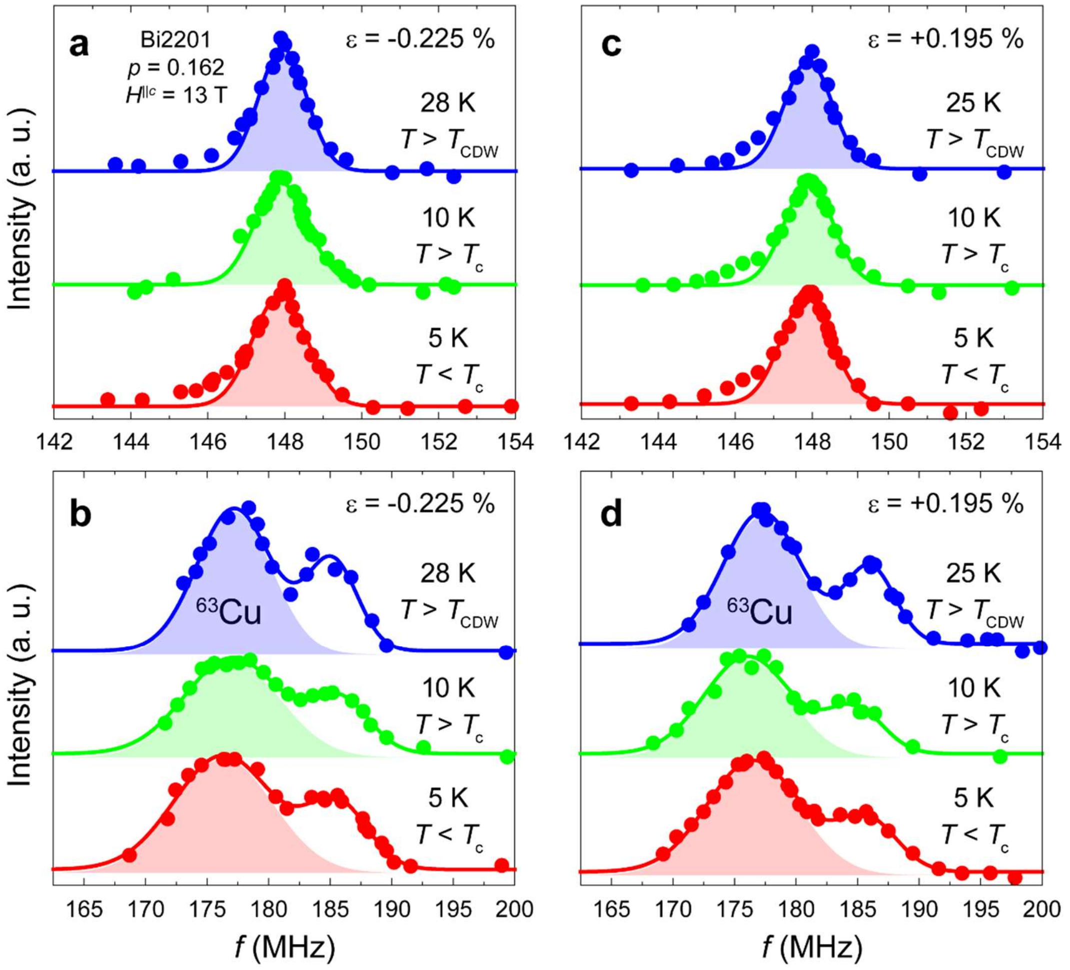

Supplementary Figure 5 | NMR spectra across T_c in the CDW ordered state. Temperature dependence of the ^{63}Cu center and satellite peaks at $\varepsilon = -0.225$ % (**a**, **b**) and $\varepsilon = +0.195$ % (**c**, **d**), respectively. Solid curves indicate the Gaussian fittings. For the center line, Cu metal is excluded. For the ^{63}Cu satellite line, two-Gaussian fits are employed to obtain the FWHM. The solid curve is the sum of the two Gaussian functions for the ^{63}Cu and ^{65}Cu satellites. Shaded area indicates the ^{63}Cu-NMR spectrum.

Supplementary Note 5

Supplementary Figure 5 shows the temperature dependence of the ^{63}Cu center and satellite spectra under compressive and tensile strains, $\varepsilon = -0.225$ % (Supplementary Fig. 5a and b) and $\varepsilon = +0.195$ % (Supplementary Fig. 5c and d), respectively. The spectral shape shows no significant difference across $T_c(\varepsilon)$ in the long-range CDW ordered state within the experimental resolution, indicating that the CDW amplitude does not change upon entering the superconducting state. Hence, the strain-induced long-range CDW order and superconductivity are coexisting. However, the NMR spectra cannot resolve whether or not the superconducting electron pairs are spatially modulated in the superconductivity coexisting with CDW, such as a pair density wave state [64].

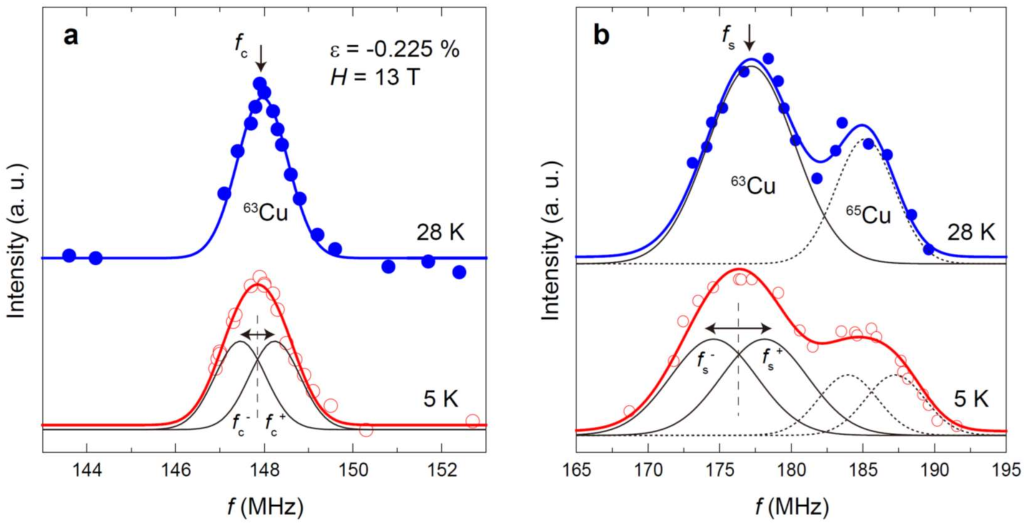

Supplementary Figure 6 | Cu-NMR spectrum analysis. Temperature dependence of the NMR center **a** and the satellite **b** lines obtained at $\varepsilon = -0.255$ % and at $H = 12.951$ T. Solid curves are Gaussian fittings to obtain the peak positions.

Supplementary Note 6

Supplementary Figure 6a and b show the temperature dependence of the ^{63}Cu center and ^{63}Cu and ^{65}Cu satellite spectra under compressive strains $\varepsilon = -0.225$ % and $H = 13$ T. The center and the satellite lines shift to lower frequency and become broad at $T = 5$ K. The peak frequency determined by the Gaussian fittings for the center line (f_c) and for the satellite line (f_s) of ^{63}Cu at $T = 28$ K are written as follows (see Methods);

$$f_c = {}^{63}\gamma(1+K)H_0 = 147.962 \text{ MHz} \cdots (1),$$
$$f_s = {}^{63}\gamma(1+K)H_0 + \nu_Q = 177.209 \text{ MHz} \cdots (2)$$

Here, $H_0 = 12.951$ T, $f_0 = {}^{63}\gamma H_0 =$ const., and $\nu_Q = 28.4$ MHz is T-independent. The origin of the peak shift at $T = 5$ K to a lower frequency is the reduction of K due to the pseudogap opening and due to the superconducting transition at $T_c = 7.2$ K.

At $T = 5$ K below T_{CDW}, assuming that they split into two equivalent spectra, namely K and ν_Q are divided into two components due to static charge distribution as $\pm\delta K$ and $\pm\delta\nu_Q$, respectively. The results of fitting the ^{63}Cu and ^{65}Cu spectra are shown in the figure. The solid and dotted curves represent the Gaussian fitting curves and their sum, respectively. The line width of each spectrum is fixed to the value at $T = 28$ K, $\delta\nu_Q$ for ^{65}Cu is determined by the factor of $^{65}Q/^{63}Q$, which is the ratio of the quadrupole moments of ^{63}Cu and ^{65}Cu, and the only free parameter is the peak frequency. The fitting results are in good agreement with the experimental data.

The peak frequency determined by the Gaussian fittings for the center line ($f_c^{\pm}$) and for the satellite line ($f_s^{\pm}$) of ^{63}Cu at $T = 5$ K are written as follows;

$$f_c^{+} = {}^{63}\gamma(1+K+\delta K)H_0 = 148.236 \text{ MHz} \cdots (3)$$

$f_c^- = {}^{63}\gamma(1 + K - \delta K)H_0 = 147.461$ MHz $\cdots$ (4)
$f_s^+ = {}^{63}\gamma(1 + K + \delta K)H_0 + \nu_Q + \delta\nu_Q = 178.128$ MHz $\cdots$ (5)
$f_s^- = {}^{63}\gamma(1 + K - \delta K)H_0 + \nu_Q - \delta\nu_Q = 174.578$ MHz $\cdots$ (6)

Finally, we obtain $\delta K = 0.00265$ and $\delta\nu_Q = 1.39$ MHz, respectively. From the relation, $\nu_Q = 22.0 + 39.6p$ [29], we find that these values are produced by the carrier distribution $\delta p = 0.035$ at the Cu-site due to the long-range CDW order.

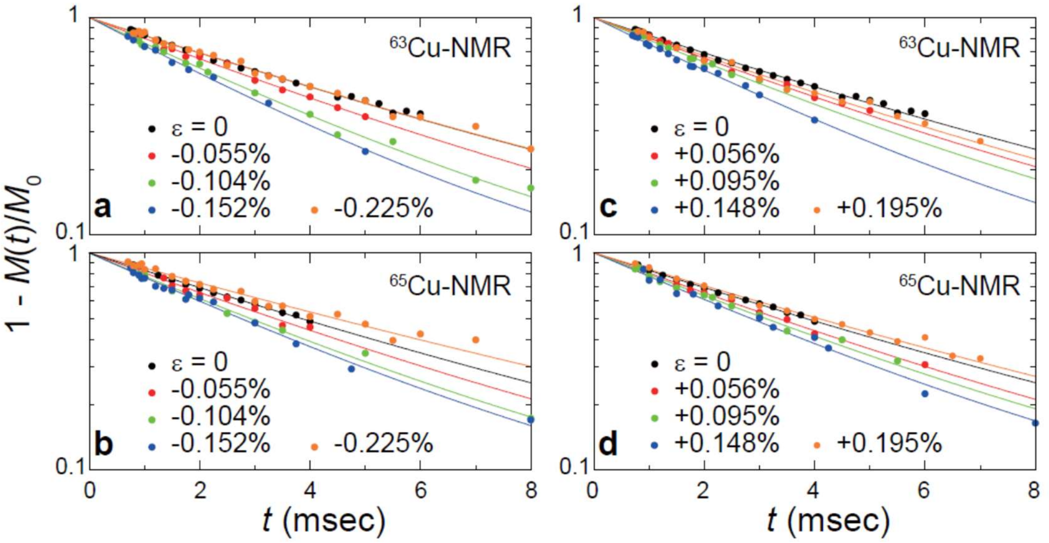

Supplementary Figure 7 | Recovery curves to obtain T_1. Compressive and tensile strain dependence of the recovery curves for ^{63}Cu (**a**, **c**) and for ^{65}Cu (**b**, **d**) obtained at T = 10 K. Solid curves are the results of the fitting by the theoretical function, $1 - M(t)/M_0 = 0.9 \exp(-6t/T_1) + 0.1 \exp(-t/T_1)$.

Supplementary Note 7

To obtain T_1, the time dependence of the spin-echo intensity after the saturation of the nuclear magnetization M (recovery curve) was fitted by the theoretical function [76], $1 - M(t)/M_0 = 0.9 \exp(-6t/T_1) + 0.1 \exp(-t/T_1)$ where M_0 and $M(t)$ were the magnetization in the thermal equilibrium and at a time t after a single saturating pulse. Supplementary Fig. 7 shows the recovery curves for ^{63,65}Cu obtained at T = 10 K under compressive (Supplementary Fig. 7a and b) and tensile (Supplementary Fig. 7c and d) strains, respectively. Our previous measurements have shown that the recovery curve has a single T_1 component [44]. In this study, the sample size was tiny and the signal-to-noise ratio was poor. Therefore, we fitted the theoretical curve to the data up to 90 % recovery, where the accuracy of the data can be trusted, and determined T_1 under strain.
